# Disruption of Pseudomonas aeruginosa quorum sensing influences biofilm formation without affecting antibiotic tolerance

**DOI:** 10.1099/mic.0.001557

**Published:** 2025-04-25

**Authors:** Elvina Smith, Andrew Matthews, Edze R. Westra, Rafael Custodio

**Affiliations:** 1 Environment and Sustainability Institute, Biosciences, University of Exeter, Penryn Campus, Penryn TR10 9FE, UK; 2 Instituto de Biologia Molecular e Celular, Universidade do Porto, Porto, Portugal; 3 i3S, Instituto de Investigação e Inovação em Saúde, Universidade do Porto, Porto, Portugal

**Keywords:** antibiotic resistance, autolysis, biofilm, *Pseudomonas aeruginosa*, quorum sensing

## Abstract

The opportunistic bacterial pathogen *Pseudomonas aeruginosa* is a leading cause of antimicrobial resistance-related deaths, and novel antimicrobial therapies are urgently required. *P. aeruginosa* infections are difficult to treat due to the bacterium’s propensity to form biofilms, whereby cells aggregate to form a cooperative, protective structure. Autolysis, the self-killing of bacterial cells, and the bacterial cell-to-cell communication system, quorum sensing (QS), play essential roles in biofilm formation. Strains of *P. aeruginosa* that have lost the *lasI/R* QS system commonly develop in patients, and previous studies have characterized distinctive autolysis phenotypes in these strains. Yet, the underlying causes and implications of these autolysis phenotypes remain unknown. This study confirmed these autolysis phenotypes in the PA14 QS mutant strains, Δ*lasI* and Δ*lasR*, and investigated the consequences of QS loss and associated autolysis on biofilm formation and antibiotic susceptibility. QS mutants exhibited delayed biofilm formation but ultimately surpassed the wild-type (WT) in biofilm mass. However, the larger biofilm mass of the QS mutants was not reflected in higher live-cell numbers, indicating an altered biofilm structure. Nevertheless, QS mutant biofilms were not more susceptible to antibiotics than the WT. Artificial supplementation of Δ*lasI* with a QS signal molecule (autoinducer) restored the strain’s QS system without the associated costs of QS, enabling Δ*lasI* to achieve higher pre-treatment and post-treatment live-cell numbers. Overall, the lack of QS functioning was not detrimental to biofilm antibiotic tolerance, though the artificial disruption of QS may reduce the advantages of QS mutants within *in vivo* mixed-strain populations. Much remains to be understood regarding the regulation and induction of the autolysis phenotypes observed in these strains, and future research to fully elucidate the control and consequences of autolysis may offer potential for novel antimicrobial therapies.

## Data Availability

The experimental data used to support the findings of this study are available from the corresponding author upon request.

## Introduction

Antimicrobial resistance (AMR) is a major global health threat, responsible for an estimated 1.27 million deaths in 2019 and predicted to cause 10 million deaths annually by 2050 [1,5]. *Pseudomonas aeruginosa* is an opportunistic pathogen and is among the top six AMR-associated bacteria, responsible for over 250,000 deaths in 2019 [3,6]. *P. aeruginosa* poses a particular risk to immunocompromised individuals, such as those with cystic fibrosis (CF), and is considered a priority pathogen by World Health Organization for requiring novel antimicrobial therapies and strategies [7,8]. A greater understanding of *P. aeruginosa* evolution and ecology is fundamental to developing such strategies [9,10].

The propensity of *P. aeruginosa* to form biofilms makes it particularly difficult to control and treat [11,14]. Biofilms are structured aggregations of microbial cells encased within a self-produced polymer matrix [15,17]. The fortified structure of biofilms enhances bacterial tolerance to environmental stressors, immune responses and antimicrobial agents [6,17, 18]. Within clinical settings, biofilms are notorious for causing persistent infections and can be up to 1,000 times more resistant to antimicrobials than planktonic cells [6,19,22]. Biofilms represent a form of bacterial cooperation, consisting of cells that make up a differentiated structure that functions collectively [6,17, 23]. Autolysis (the self-killing of bacterial cells) is a fundamental process in biofilm formation and maintenance [24,26]. During early biofilm development, autolysis releases extracellular DNA (eDNA) necessary for the initial adhesion of cells, with eDNA acting as an essential cell-to-cell connecting compound of the biofilm matrix [15,26]. The release of eDNA during autolysis can occur through multiple mechanisms, including the expression of phage-like endolysins encoded within the R-/F-pyocin locus and the AlpR-regulated programmed cell death pathway [27,28]. This fortification of the biofilm matrix, via autolysis, limits the diffusion of antimicrobials into the biofilm, resulting in increased resistance [29].

The bacterial communication system, quorum sensing (QS), plays a key role in biofilm formation and autolysis regulation [26,30]. In a fully functioning QS system, bacteria can produce and receive diffusible signal molecules called autoinducers, allowing bacterial populations to regulate gene expression in response to cell density fluctuations [12,23, 31, 32]. Studies on *P. aeruginosa* have indicated that QS and autolysis regulation are highly interlinked [11,33]. Mutants of the PAO1 strain that overproduce the Pseudomonas quinolone signal (a QS molecule) have displayed pronounced autolysis at the centre of biofilms [33]. In the PA14 strain, the lack of a functional QS system has also been associated with increased autolysis [34]. The molecular mechanisms underpinning the autolysis phenotypes of *P. aeruginosa* QS mutants are not fully understood, though suggestions include the activation of a prophage, whose genes are highly upregulated during biofilm growth [33]. Prophage genes have demonstrated an 83.5-fold increase in activation during *P. aeruginosa* biofilm development, compared to expression in planktonic cells [35]. It has been suggested that manipulation of QS can help to remove or prevent the formation of *P. aeruginosa* biofilms. For example, QS inhibitors could be used to disrupt biofilms, either by reducing QS-induced autolysis and subsequent biofilm promotion or by inducing autolysis to enhance antibiotic penetration [12,30, 33, 36]. While these previous studies have demonstrated a causal relationship between cell-to-cell signalling and autolysis, the effect of QS-dependent autolysis suppression or promotion on biofilm formation and antibiotic tolerance has not been systematically investigated.

QS-deficient *P. aeruginosa* mutants frequently arise in clinical and environmental settings [31,37]. In CF patients, QS mutants have become a marker of early-stage chronic infection [34]. These include mutants that have lost the functioning of the *lasI/R* genes [31]. The *lasI* gene is an acyl-lactone synthase, responsible for the production of the QS signal molecule (autoinducer) *N*-3-oxododecanoyl homoserine lactone (C12-HSL). The *lasR* gene is a transcriptional activator, for which the cognate inducer is C12-HSL. This means that *P. aeruginosa* mutants that have lost functioning of the *lasI* gene (Δ*lasI*) are ‘signal-negative’ mutants, as they cannot produce these signal molecules but retain the ability to receive them [31]. By contrast, mutants that have lost the *lasR* gene (Δ*lasR*) function can no longer receive the signal and are referred to as ‘signal-blind’ [31]. Although these mutants naturally arise and thrive within *P. aeruginosa* populations, when grown as biofilms, Δ*lasI* and Δ*lasR* have demonstrated distinctive autolysis phenotypes not observed in the wild-type (WT) [13,34, 38]. These lysis phenotypes present as holes/plaques in the bacterial lawn and have been observed in laboratory and clinical strains [12,13, 37]. The full effects of these autolysis phenotypes on biofilm formation and maintenance, especially regarding antibiotic susceptibility, are unknown.

This study examined the relationship between these autolysis phenotypes and the loss of QS function on biofilm formation and antibiotic susceptibility using QS mutant strains (Δ*lasI* and Δ*lasR*) of PA14. To confirm if this autolysis phenotype was linked explicitly with the loss of QS ability, phenotypes were compared with and without the artificial supplementation of an autoinducer C12-HSL. The impact of QS deficiency and implicated autolysis phenotypes on bacterial growth and biofilm formation was examined. Finally, biofilm treatment with antibiotics was compared across strains, with and without the supplementation of autoinducer, to determine if the loss of *lasI/R* function impacts tolerance to antibiotic treatment. It was hypothesized that (1) if the autolysis phenotypes observed in Δ*lasI* and Δ*lasR* are caused by the lack of a functioning QS system, the supplementation of bacteria with C12-HSL should restore the WT phenotype in the signal-negative mutant (Δ*lasI*), but not the signal-blind mutant (Δ*lasR*); (2) if QS cooperation and QS regulation of autolysis are important for PA14 growth in the social context of biofilms, then QS mutants should display growth differences compared to the WT when grown as a biofilm; (3) given that biofilms are more resistant to antibiotics, if QS and QS regulation of autolysis affect biofilm formation and maintenance (a) biofilms of WT and QS mutants will display different tolerances to antibiotics and (b) the supplementation with C12-HSL will affect antibiotic treatment response for Δ*lasI*, but not WT or Δ*lasR*.

## Methods

### Bacterial strains

The *P. aeruginosa* laboratory strain UCBPP-PA14 [39] was the WT strain used in this study. The UCBPP-PA14 mutants Δ*lasI* and Δ*lasR* [40] were used as QS mutant strains, lacking the respective *lasI* and *lasR* QS genes required for a fully functioning QS system. These strains were selected for their specific lack of ability to produce (Δ*lasI*) and receive (Δ*lasR*) the QS signal molecule (autoinducer) C12-HSL.

Strains were stored in LB (Miller formulation) broth supplemented with 30% (v/v) glycerol at −80 °C. Pre-cultures (used to inoculate the primary experimental cultures) were grown on solid LB agar plates overnight, from which single colonies were used for inoculation. All bacterial cultures were grown in incubators at 37 °C. Planktonic cultures were grown under shaking conditions (180 r.p.m.).

### Planktonic growth assays

Planktonic growth assays were conducted to check for strain-dependent growth differences when grown as free-living bacteria instead of biofilms. Aliquots containing 200 µl of the diluted overnight culture (1:100 in LB broth) were added to each well of a 96-well plate. Each strain had nine replicate wells, and nine control wells were filled with 200 µl of LB. Each well was topped with 20 µl of mineral oil to prevent evaporation during incubation inside the plate reader. Optical density (OD) measurements were conducted in the BioTek Synergy 2 plate reader. Cultures were incubated at 37 °C with agitation, and OD_600_ readings were taken every 15 min for 24 h.

### Autolysis phenotypes of WT and QS mutant strains

Autolysis phenotypes were visualized on solid LB agar by spotting 5 µl of overnight culture (diluted 1:100 in LB broth) onto LB agar plates. Single strains were spotted individually and side-by-side (i.e. WT with Δ*lasI*; WT with Δ*lasR*; Δ*LasI* with Δ*lasR*). Single strains were also spotted on LB agar plates supplemented with 2 µM ml^−1^ of C12-HSL autoinducer. For each treatment, three replicates were plated. Plates were incubated overnight at 37 °C, and bacterial colonies were imaged using the Azure Imaging System (Azure Biosystems).

To confirm that the observed autolysis phenotypes correlated with cell death, colony-forming units (c.f.u.) of entire colonies and the centre of colonies (most of the observed autolysis occurring within the centre of colonies) were quantified over a 72 h period. To do this, 5 µl aliquots of diluted culture for each strain were spotted on solid LB agar plates to be grown for 24, 48 and 72 h. Each strain had six replicates per time point and sample type (whole colonies and centre of colonies). At specific time points, entire colonies or the centre of colonies (collected and standardized using the wide end of a 1 ml pipette tip punched through the agar) were harvested and re-suspended in 1 ml of phosphate-buffered saline (PBS). Re-suspended cells were serially diluted, and 5 µl aliquots of each dilution were spotted onto LB agar plates. Plates were incubated at 37 °C until colonies could be counted. Colony counts were used to quantify the number of live cells for each replicate and sample type at each time point.

### Biofilm formation assay using crystal violet method

Biofilm formation was quantified using the crystal violet (CV) method, as previously reported by O’Toole [41]. Briefly, overnight cultures of each strain were diluted 1:100 in M63 medium (1×), supplemented with magnesium sulphate (1 mM), glucose [0.2% (w/v)] and casamino acids [0.5% (w/v)]. Preliminary experiments to compare biofilm formation in LB broth and supplemented M63 medium found that the latter promoted further biofilm formation for each PA14 strain, consistent with previous findings [41]; therefore, it was used for all biofilm assays in this study. This assay was performed for biofilms of each strain, with autoinducer (C12-HSL) supplementation and without. Biofilm formation was quantified for biofilms grown for 24, 48 and 72 h.

Wells of 96-well microtitre plates were inoculated with 100 µl of 1:100 diluted culture. Plates contained six replicate wells per strain, treatment and growth time. For biofilms supplemented with autoinducer supplementation, C12-HSL was added to the diluted cultures to create a final concentration of 2 µM ml^−1^. Each plate included six control wells, containing only supplemented M63 medium. Plates were sealed with Parafilm and incubated (static) at 37 °C for 24, 48 or 72 h.

After incubation, biofilms were stained with CV. Briefly, planktonic cells and medium were removed by upending the plate and shaking out the liquid. Plates were then gently submerged in water (and lightly tapped to remove any air trapped within wells) before the water was shaken out. This washing step was repeated to remove any remaining medium and unattached (non-biofilm) cells before staining. Once washed, 125 µl of 0.1% (w/v) CV solution (in water) was added to each well and plates were incubated at room temperature for 15 min. Following incubation, plates were submerged and rinsed in water four times, with plates vigorously shaken out between washes. After washing, plates were left inverted until dry (1–2 h). Once dried, the CV was solubilized by adding 125 µl of 30% (v/v) acetic acid to each well and incubated for 15 min. For each well, the solubilized CV was transferred into a new microtitre plate. A plate reader quantified absorbance at 550 nm. Six acid blank control wells, each containing 125 µl of acetic acid, were used to adjust the CV OD value to exclude any interference of the acid.

### Biofilm live cells quantification assay

Colony-forming units per ml (c.f.u. ml^−1^) of biofilms were quantified to determine the number of live cells within biofilms. This assay was performed for biofilms of each strain over a 72 h period, with autoinducer (C12-HSL) supplementation and without. The c.f.u. were quantified for biofilms grown for 24, 48 and 72 h, with six replicates per strain, treatment and growth time.

Biofilms were set up in accordance with the biofilm formation assay. After incubation, the liquid culture was removed and wells were washed twice with 200 µl PBS to remove all planktonic cells. This was to ensure that only cells that formed biofilms were treated and quantified. Next, the bacterial cells were suspended and mixed in 100 µl PBS, with all biofilm thoroughly scraped off well walls. This suspension of biofilm cells was then serially diluted and plated, with 5 µl of each dilution spotted onto LB agar plates. These plates were incubated at 37 °C overnight and c.f.u.s were counted.

### Biofilm antibiotic treatment and live-cell quantification

The c.f.u. ml^−1^ of biofilms (with and without autoinducer supplementation) after 72 h of growth were used to investigate strain-dependent differences in the response to antibiotic exposure. Here, biofilm live-cell quantification was performed with four different treatment types: untreated, or in the presence of either chloramphenicol (Chl) 30 µg ml^−1^, tetracycline (Te) 30 µg ml^−1^ or carbenicillin (Carb) 25 µg ml^−1^. Antibiotic concentrations were based on the *P. aeruginosa* minimum inhibitory concentrations (MICs) used in Dimitriu *et al.* [42], unless stated otherwise. These antibiotics were selected to represent different bacteriostatic (Chl, Te) and bactericidal (Carb) effects on *P. aeruginosa*. The c.f.u. ml^−1^ of biofilms (with and without autoinducer supplementation) after 48 and 72 h of growth were used to investigate strain-dependent differences in the response to antibiotic exposure.

Here, biofilms were prepared as described in the biofilm live-cell quantification assay. Biofilms due to incubate for 72 h were removed from the incubator after 48 h of growth and washed once with PBS before wells were refilled with supplemented M63 medium (with/without autoinducer supplementation), containing the relevant dilution of antibiotics for each antibiotic treatment type. Untreated biofilm wells were filled with supplemented M63 medium only (with/without autoinducer supplementation). Once refilled, plates were incubated for another 24 h (total 72 h of growth) before live cells within biofilms were quantified as described in the biofilm live-cell quantification assay.

This assay was repeated with antibiotic treatments at ten times the concentration of PA14 planktonic MIC [42].

### Confocal microscopy

Morphology of each strain’s biofilm was examined following growth on glass slides. Sterile glass slides were statically incubated at 37 °C for 24 h, partially submerged in 50 ml Falcon tubes containing 20 ml of supplemented M63 medium inoculated with 1:100 dilutions of overnight cultures of each strain. Following incubation, slides were removed and cleaned on one side prior to staining.

Biofilms were stained with Invitrogen’s BacLight bacterial viability and counting staining kit, comprising SYTO 9 dye at 3.34 mM with propidium iodide at 20 mM. Using this stain to assay cell viability, dye is taken up by cells selectively depending on cell membrane integrity, making live and viable bacterial cells visible with a green fluorescence (SYTO 9) and non-viable or dead cells visible with a red fluorescence (propidium iodide). Images were acquired using a confocal fluorescence microscope (Leica AF6000) set up as follows: objective×20 plan apochromatic; SYTO9 excitation wavelength 488 nm, emission wavelength 493–547 nm; propidium iodide excitation wavelength 552 nm, emission wavelength 567–725 nm. Processing and analysis of images was conducted using Leica software (LAS X version 3.5.1.18803).

### Statistical analysis

Statistical analyses were conducted with an alpha level of 0.05 using R Core Team [43]. Data were analysed using linear models and multiple linear regression. Non-significant factors and interactions between factors were removed sequentially using backward elimination. The significance level of each factor was determined by comparing the final model with and without the factor of interest using F-tests. Diagnostic plots (residuals vs. fitted, Q-Q, residuals vs. leverage and scale-location plots) were used to check model fit and that the assumptions of linear regression were not violated. Pairwise comparisons were performed using the emmeans package [44], with significance determined using Tukey-adjusted *P* values for significant model terms. These adjusted *P* values are reported throughout the results section.

For planktonic growth, represented by OD_600_ measurements over a 24 h period, data were logged (log_10_), and results were analysed using the Growthcurver R package [45]. To determine if strains grew significantly differently, the area under the logged curves of strains was compared using a one-way ANOVA, with nine biological replicates per strain.

## Results

This study aimed to further understand the autolysis phenotypes previously observed in the PA14 QS mutant strains, Δ*lasI* and Δ*lasR*, and investigate the consequences of QS loss and associated autolysis on biofilm formation and antibiotic susceptibility.

### QS interference promotes PA14 autolysis in static conditions

**Fig. 1.**
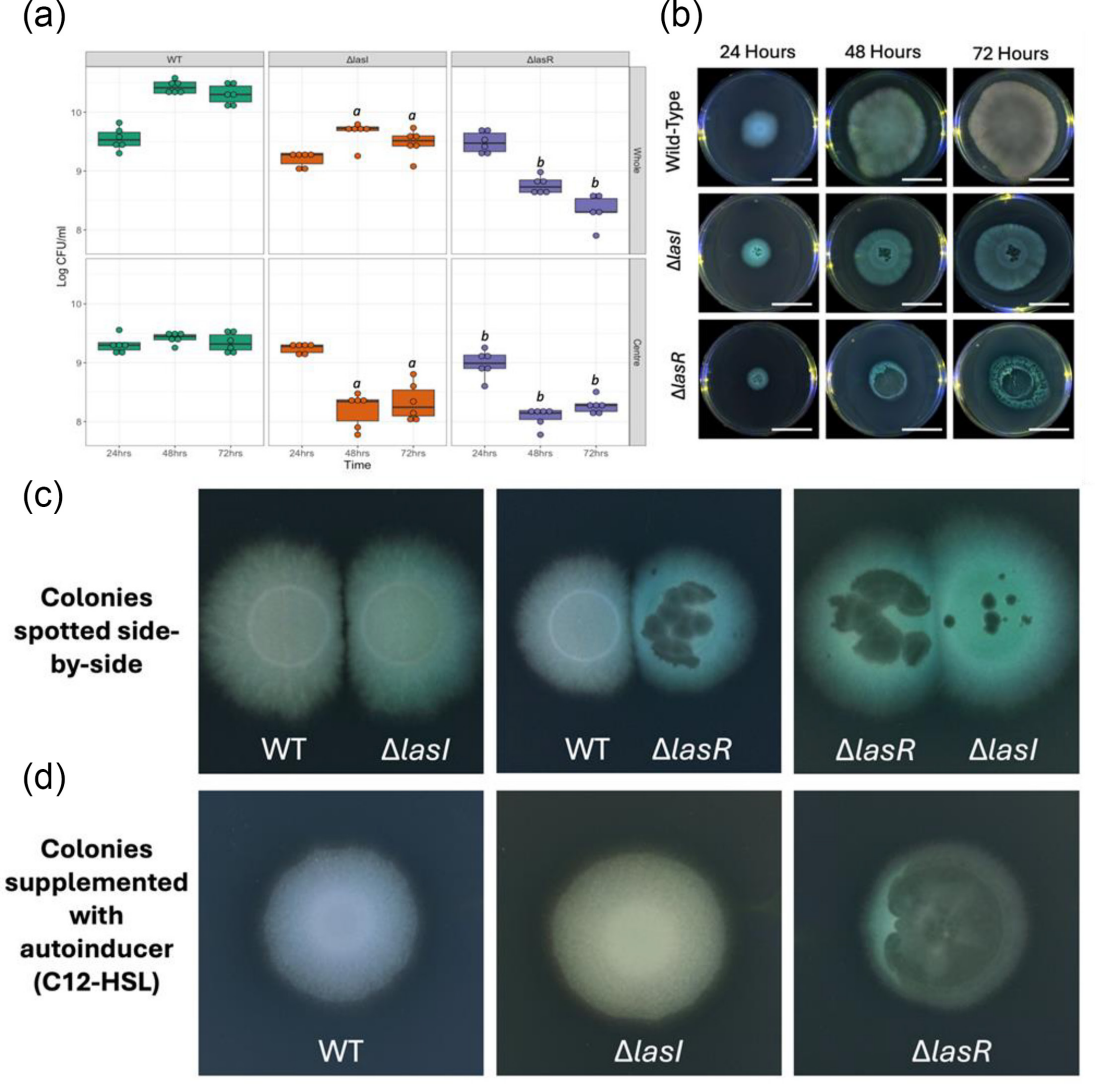
*P. aeruginosa* PA14 WT and QS mutants (*ΔlasI* and *ΔlasR*) autolysis phenotypes and c.f.u. when grown on solid medium. (**a**) The c.f.u. ml^−1^ of entire colonies (whole) and the centres of colonies (centre), grown on solid medium after 24, 48 and 72 h of growth. Box plots show the median and interquartile range, with individual data points plotted (*N*=6 replicates per strain, time point and sample type). The c.f.u. ml^−1^ values are presented on the log_10_ scale. Significant differences (Tukey-adjusted *P* value: *P*<0.05) identified between the WT and QS mutants at individual growth times are marked with *a* for Δ*lasI* and *b* for Δ*lasR*. (**b**) Photographs of PA14 WT and QS mutants (Δ*lasI* and Δ*lasR*) autolysis phenotypes after 24, 48 and 72 h of growth. Colonies were produced by spotting 5 µl of liquid diluted overnight culture onto solid medium to be grown and incubated at 37 °C and photographed. Scale bars represent 3 cm in size. (**c**) Strains were spotted side-by-side to be incubated overnight at 37 °C and photographed. (**d**) Colonies of single-strain cultures were spotted onto solid LB agar supplemented with the QS autoinducer C12-HSL to be incubated overnight at 37 °C and photographed.

To confirm the different autolysis phenotypes of the QS mutants, strains were grown on solid medium and compared. When grown as individual strains, within 24 h of growth, QS mutants developed visible zones of autolysis in the bacterial lawn that were not visible in the WT, consistent with previous studies [34] (Fig. 1b). Lysis zones presented as small, numerous holes in the bacterial lawn of Δ*lasI* and large plaques spreading from the centre to the edges of the entire colony in Δ*lasR* (Fig. 1b). These zones of lysis increased in size over a 72 h period (Fig. 1b). To determine if these autolysis phenotypes resulted from a non-functioning QS system, the development of these phenotypes in QS mutants was compared in the presence of the WT or supplemented with autoinducer. When grown alongside the WT, Δ*lasI* did not exhibit any visible zones of autolysis (Fig. 1c); by contrast, Δ*lasR* still exhibited autolysis (Fig. 1d). Autoinducer (C12-HSL) supplementation restored the WT phenotype, of no visible autolysis, in Δ*lasI* but did not change the autolysis phenotype of Δ*lasR* (Fig. 1d). Altogether, these data confirmed that the *las* system is responsible for the observed autolysis. Additionally, Δ*lasR*-dependent lysis was not altered by the supplementation of autoinducer, as would be expected by a signal-blind mutant.

To confirm that the lysis phenotypes observed correlated with cell death, c.f.u. ml^−1^ (representing the number of living cells) of strains grown on solid medium were quantified for entire colonies and the centre of colonies, where autolysis was typically observed. Throughout a 72 h period, the number of colonies differed dependent on strain when whole colonies (F_4,44_=20.733, *P*<0.001) or the centre of colonies (F_4,45_=8.600, *P*<0.001; Fig. 1a) were sampled. After 24 h of growth, c.f.u. ml^−1^ of whole colonies was not significantly different (Tukey-adjusted *P* value 24 h: Δ*lasI P*=0.612, Δ*lasR P*=0.973; Fig. 1a). However, after 48 and 72 h of growth, both QS mutants have shown reduced c.f.u. ml^−1^ compared to the WT (Tukey-adjusted *P* value: Δ*lasI P*<0.001, Δ*lasR P*<0.001 for 48 and 72 h; Fig. 1a), corresponding with the increased amounts of lysis observed within the QS mutant colonies (Fig. 1b). When sampling only the centre of colonies, the lysis that had occurred by 24 h in Δ*lasR* had reduced the c.f.u. ml^−1^ compared to the WT and Δ*lasI* strains (Tukey-adjusted *P* value 24 h: WT *P*=0.001, Δ*lasI P*=0.032; Fig. 1a). After 48 and 72 h of growth, the lysis occurring within the centres of colonies of QS mutants produced a substantial reduction in c.f.u. ml^−1^ of both QS mutants compared to the WT (Tukey-adjusted *P* value: Δ*lasI P*<0.001, Δ*lasR P*<0.001 for 48 and 72 h; Fig. 1a). This reduction in c.f.u. for the QS mutants corresponded with the increase in size of the lysis plaques over 72 h that were not observed in the WT. This indicates the lysis phenotypes observed in the QS mutants corresponded with increasing cell death and reduced growth.

### Disruption of *las* system does not impact planktonic growth

**Fig. 2.**
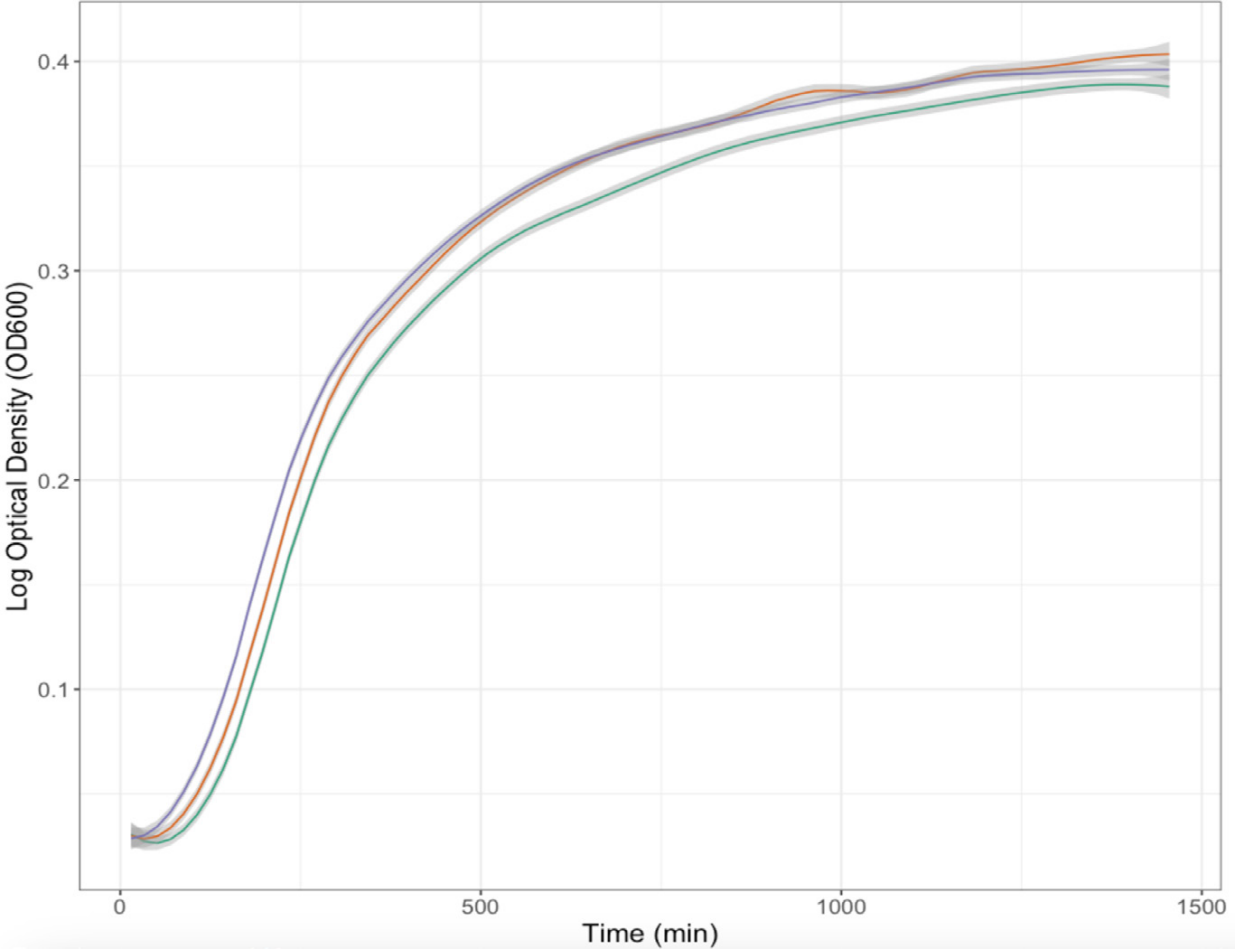
Increase in OD_600_ of *P. aeruginosa* PA14 planktonic cultures over time (minutes) for WT and QS mutant (Δ*lasI* and Δ*lasR*) strains. OD increase represents bacterial growth over time for WT (green), Δ*lasI* (orange) and Δ*lasR* (purple) strains. OD_600_ measurements are represented on the log_10_ scale. Measurements were taken every 15 min within a 24 h period. Lines display conditional means (OD_600_ dependent on time) of nine replicates per strain, with shaded areas representing standard error.

To determine if any strain-dependent growth differences between the WT and QS mutants were exclusive to the biofilm or static conditions, growth in planktonic suspension was compared. Over a 24 h period, the bacterial density (OD_600_) of each strain significantly increased over time (F_2,288_=6066.700, *P*<0.001; Fig. 2). The QS mutant strains demonstrated no significant differences to the WT during this period, with no significant effect of strain on bacterial density (OD_600_) (F_2,228_=1.014, *P*=0.364) or rate of growth over time (interaction between time and strain on bacterial density: F_2,282_=4.420, *P*=0.091) (Fig. 2). Thus, the QS mutants exhibited no differences from the WT when grown as free-living bacteria.

### QS deficiency affects biofilm formation dynamics

**Fig. 3.**
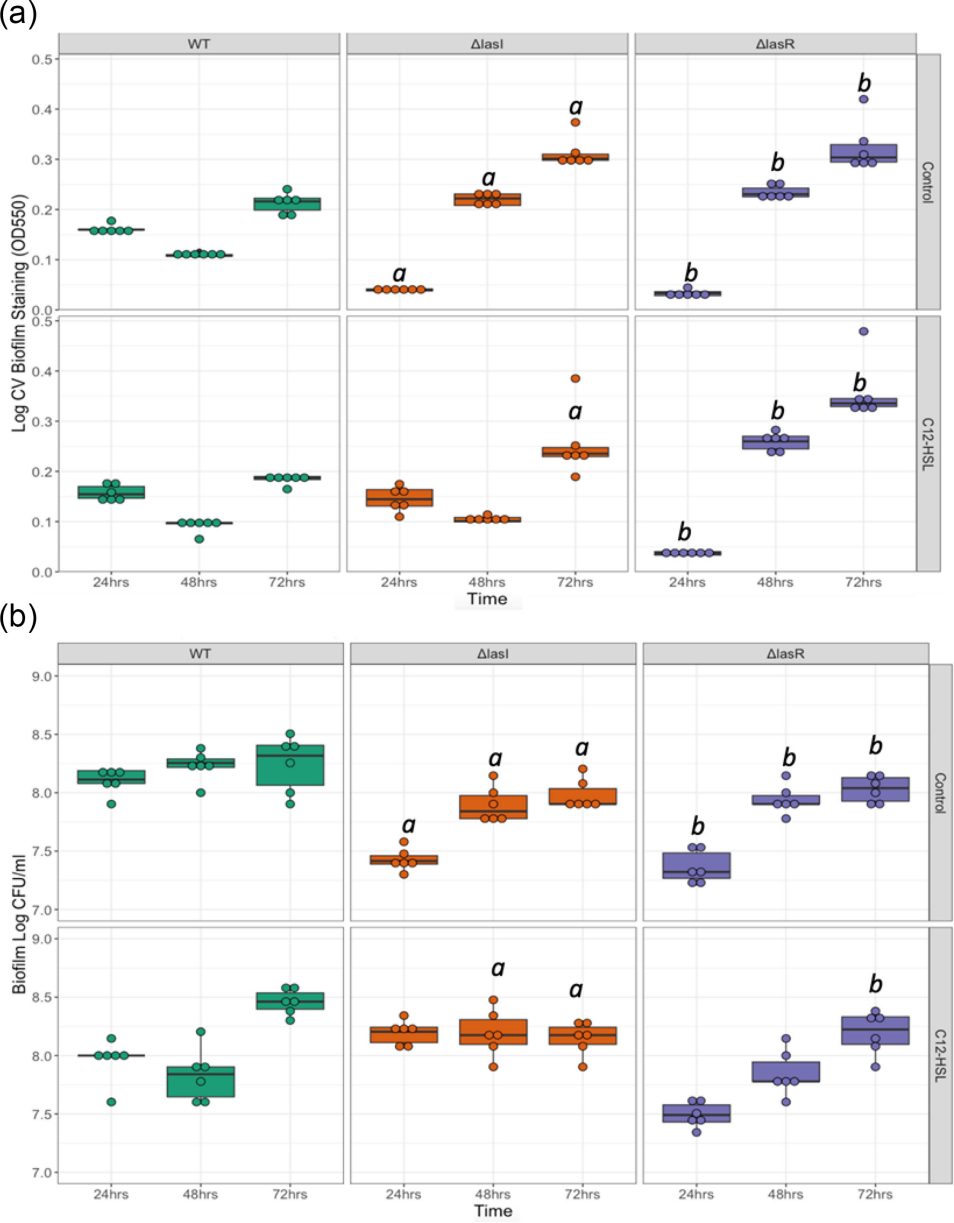
Biofilm formation of *P. aeruginosa* PA14 WT and QS mutants (Δ*lasI* and Δ*lasR*) with and without autoinducer supplementation, measured by CV staining and c.f.u. ml^−1^. Biofilms of each strain were grown in wells of microtitre plates for 24, 48 and 72 h. C12-HSL wells were supplemented with C12-HSL autoinducer at a 2 µM ml^−1^ concentration. Box plots show the median and interquartile range, with individual data points plotted (*N*=6 replicates per strain, time point and treatment). Values are presented on the log_10_ scale. Significant differences (Tukey-adjusted *P* value: *P*<0.05) identified between the WT and QS mutants at individual growth times are marked with *a* for Δ*lasI* and *b* for Δ*lasR*. (**a**) Biofilms were stained using CV and the OD of solubilized CV measured at 550 nm. (**b**) The c.f.u. ml^−1^ recovered from biofilms.

Since QS disruption was associated with increased autolysis and formation of plaques in bacterial colonies, next, the ability of QS mutant strains to form biofilms was assessed via CV staining at 24, 48 and 72 h. Biofilms were also compared with and without supplementation of the *lasI/R* signalling molecule C12-HSL to determine if strain-dependent differences were specific to the lack of QS-dependent signalling. The degree of biofilm formation was considerably different for each strain, with the level of CV staining significantly differing depending on strain over time (F_4,94_=35.361, *P*<0.001) (Fig. 3a). The WT showed superior biofilm formation at 24 h of growth (WT 24 h Tukey-adjusted *P* value: Δ*lasI P*<0.001 and Δ*lasR P*<0.001). This pattern subsequently reversed, with QS mutant biofilms exhibiting increased biofilm mass at 48 and 72 h of growth (WT 48 h Tukey-adjusted *P* value: Δ*lasI P*<0.001 and Δ*lasR P*<0.001; WT 72 h Tukey-adjusted *P* value: Δ*lasI P*<0.001 and Δ*lasR P*<0.001) (Fig. 3a). Strikingly, though biofilm formation in the QS mutants was initially slower than in the WT, by 72 h the QS mutants had surpassed the WT in biofilm formed. When Δ*lasI*’s QS function was restored with the supplementation of autoinducer, Δ*lasI* produced similar amounts of biofilm to the WT at 24 and 48 h (significant interaction between strain and autoinducer supplementation: F_2,94_=4.190, *P*=0.018; Δ*lasI* C12-HSL Tukey-adjusted *P* value to WT C12-HSL 24 h *P*=0.910 and 48 h *P*=0.9301), though still exceeded WT biofilm after 72 h of growth (Δ*lasI* Tukey-adjusted *P* value: WT 72 h *P*<0.001).

Although CV staining indicated the QS mutants formed more biofilm than the WT, this method stains both live and dead cell components within the biofilm [46]. Therefore, bacteria recovered from 24, 48 and 72-h-old biofilms were compared between strains to determine if this increased biofilm formation of the QS mutants reflected increased live-cell numbers and biofilm viability. Despite the increased CV staining observed in the QS mutants compared to the WT, this was not reflected in the number of bacteria recovered from biofilms. Though the number of bacteria recovered from biofilms did differ dependent on strain (F_4,94_=5.428, *P*<0.001), this was due to slightly higher live-cell numbers observed within WT biofilms compared to the QS mutants (WT 24 h Tukey-adjusted *P* value: Δ*lasI P*<0.001 and Δ*lasR P*<0.001; WT 48 h Tukey-adjusted *P* value: Δ*lasI P*=0.001 and Δ*lasR P*=0.002; WT 72 h Tukey-adjusted *P* value: Δ*lasI P*<0.001 and Δ*lasR P*=0.002) (Fig. 3b). This contrasts with the increased level of biofilm formation observed in the QS mutants when using CV staining and suggests that the larger biofilm mass formed by the QS mutants may contain a lower density of live cells compared to the WT. Indeed, increased levels of cell death and the filamentation of dead cells in the QS mutant biofilms could be observed when viewing biofilms using confocal microscopy (Fig. S2, available in the online Supplementary Material).

The effect of autoinducer supplementation only altered biofilm c.f.u. for Δ*lasI* (F_2,94_=8.960, *P*<0.001; Δ*lasI* control compared to C12-HSL supplemented Tukey-adjusted *P* value: *P*<0.001). Indeed, at 48 h of growth, Δ*lasI* biofilms achieved higher c.f.u. than the WT (Tukey-adjusted *P* value: *P*=0.007) (Fig. 3b). Thus, when Δ*lasI*’s QS function was restored with autoinducer supplementation, live cells recovered from biofilms could reach higher levels comparable with WT biofilms. Conversely, autoinducer supplementation did not affect live biofilm cell counts of Δ*lasR*, which could not receive the signal. Altogether, these results highlight the importance of *lasI/R* regulation in biofilm structural dynamics.

**Fig. 4.**
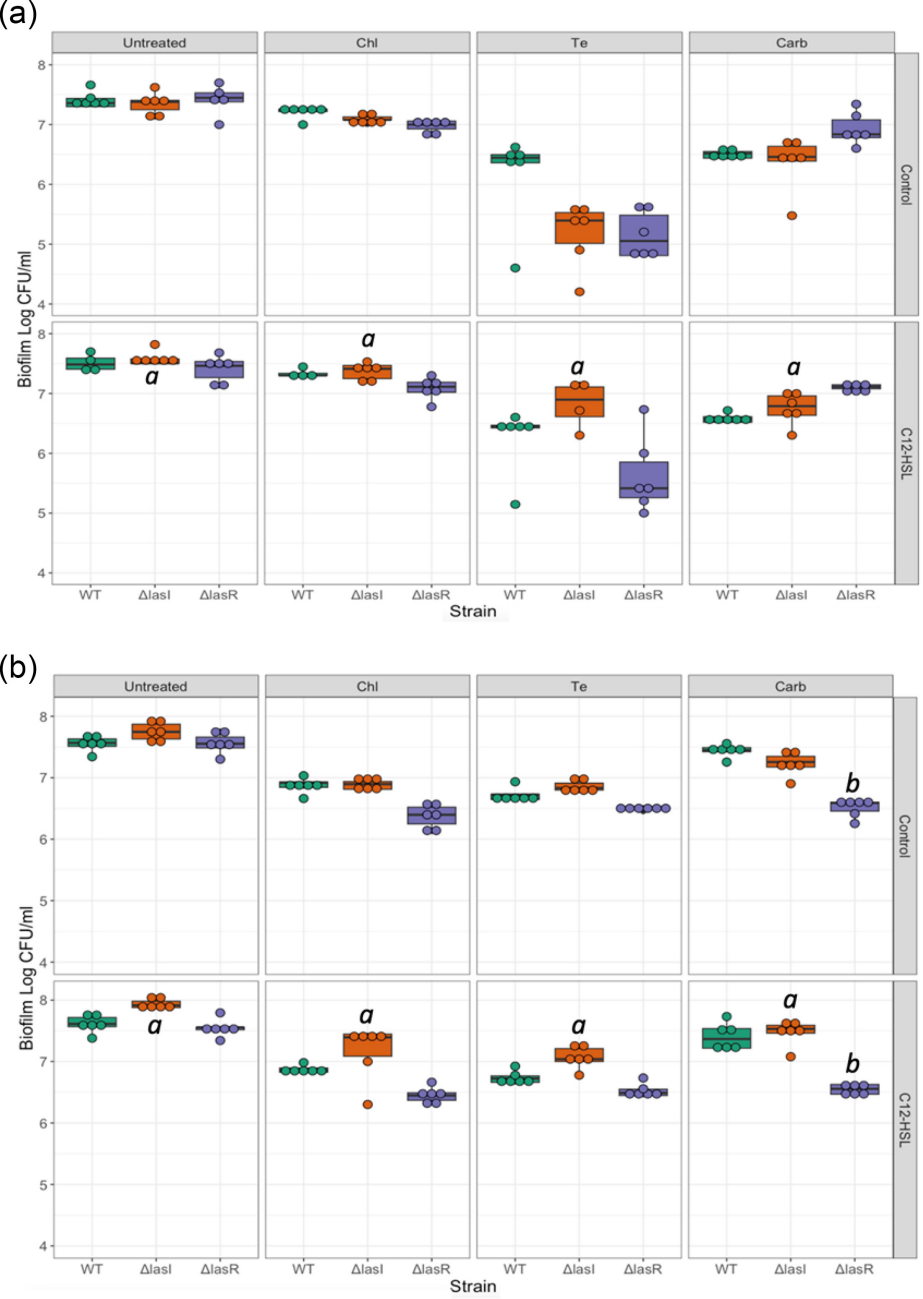
The c.f.u. ml^−1^ recovered from *P. aeruginosa* PA14 WT and QS mutant (Δ*lasI* and Δ*lasR*) biofilms, with and without the addition of autoinducer C12-HSL, for different antibiotic treatments. (**a**) Antibiotic treatments were provided at the concentration of PA14 planktonic MIC. (**b**) Antibiotic treatments were provided at ten times the concentration of PA14 planktonic MIC. Biofilms of each strain were grown for 72 h in wells of microtitre plates with four different treatment types: untreated, chloramphenicol (Chl), tetracycline (Te) and carbenicillin (Carb). In control biofilms, no autoinducer was added (*N*=6 biofilms per strain and treatment type). C12-HSL wells were supplemented with C12-HSL at a 2 µM ml^−1^ concentration (*N*=6 biofilms per strain, treatment type and C12-HSL supplementation). Values are presented on the log_10_ scale. Box plots show the median and interquartile range, with individual data points plotted. *a* indicates significant differences (Tukey-adjusted *P* value: *P*<0.05) when comparing strains with and without autoinducer for a specific antibiotic. *b* corresponds to significant differences (Tukey-adjusted *P* value: *P*<0.05) when comparing the WT and QS mutant strains within the same treatment.

To determine if the different autolysis phenotypes and biofilm-forming abilities of the QS mutants altered their susceptibility to antibiotics, biofilm growth was compared for biofilms treated with different antibiotics. To determine if any strain-dependent differences were specifically linked to QS deficiency, antibiotic-treated biofilms were tested with and without the supplementation of autoinducer. A universal result for each strain was that treated biofilms, for each antibiotic used, displayed lower live-cell numbers than untreated biofilms (Tukey-adjusted *P* value for each strain and antibiotic: *P*<0.05 for c.f.u. ml^−1^ comparing untreated and antibiotic-treated biofilms) (Fig. 4a). Notably, within biofilms without the autoinducer, there were no significant differences between strains across the antibiotic treatment types, indicating the QS mutants were no more vulnerable to the antibiotic treatments than the WT (no significant effect of strain: F_2,67_=0.985, *P*=0.379; no significant interaction between strain and antibiotic treatment type: F_2,59_=1.517, *P*=0.188; Tukey-adjusted *P* value *P*>0.05 for each strain comparison within antibiotic treatments). Similar results were observed when increasing antibiotic concentrations 10-fold. The only exception was observed in Δ*lasR* biofilms treated with carbenicillin, where c.f.u. levels were markedly lower compared to the WT (Tukey-adjusted *P* value: *P*<0.001 with and without autoinducer supplementation) (Fig. 4b). Strain-dependent differences in biofilm c.f.u. ml^−1^ only arose with the addition of autoinducer supplementation (significant interaction between strain and autoinducer addition: F_2,124_=4.859, *P*=0.009) (Fig. 4a). Here, regardless of treatment type, autoinducer supplementation of Δ*lasI* biofilms, and the consequent restoration of QS function, increased the amount of bacteria recovered when compared to Δ*lasI* biofilms with no autoinducer added (Tukey-adjusted *P* value: *P*<0.001) (Fig. 4a, b).

Overall, though biofilms of all strains exhibited decreased c.f.u. ml^−1^ compared to untreated biofilms, the QS mutant strains of PA14 were capable of growing biofilms with comparable levels of antibiotic tolerance to the WT strain.

## Discussion

This study examined the relationship between the loss of QS function on autolysis and biofilm formation in *P. aeruginosa* by comparing WT and QS mutant (Δ*lasI* and Δ*lasR*) strains of PA14. We found that QS mutants exhibited distinct colony morphologies, with zones of autolysis that were not visible in the WT, as is consistent with previous studies [13,34, 37, 38]. The QS mutants produced biofilms with increased mass, but not live-cell numbers, compared to the WT. Nonetheless, QS mutant biofilms were equally susceptible to antibiotics as WT biofilms.

These results are surprising, as autolysis is crucial in initial biofilm formation by releasing proteins, polysaccharides, nutrients and eDNA that make up the biofilm matrix [47,48,49]. While autolysis can promote biofilm formation, mutants with excessive or improperly timed autolysis can result in defective biofilms [49]. This balance highlights the importance of cell-to-cell communication and density monitoring in regulating autolysis for optimal biofilm formation [49,50].

QS mutants are common in chronic infections and are often due to mutations in signal production (Δ*lasI*) or signal sensing (Δ*lasR*). The Δ*lasI* and Δ*lasR* mutant autolysis phenotypes that we observed (Fig. 1b–d) are consistent with previous studies, with Δ*lasR* lysis zones described to have a distinct flattening caused by cell autolysis [13,34, 37, 38], although specific phenotypes can vary across strains [34].

Consistent with our observations, QS-deficient mutants of PAO1 and clinical isolates form biofilms with delayed initiation compared to the WT [51,52]. PAO1 QS mutants, including Δ*lasI*, produce dense, uniform biofilms, lacking the differentiated microcolonies and water channels observed in WT biofilms [53]. The addition of C12-HSL can restore biofilm differentiation in PAO1 Δ*lasI*, highlighting the role of QS in this process [53]. Notably, we observed considerably greater CV staining in QS mutant biofilms, a result that was not reflected in the live-cell numbers (Fig. 3). This pattern persists whether the media remains unchanged throughout the experiment or is refreshed daily (Fig. S1). As CV stains the bacteria within the biofilm and the surrounding biofilm matrix [54], the increased CV staining of Δ*lasI* and Δ*lasR* may reflect elevated autolysis and enhanced matrix production. These increased levels of cell death in the QS mutant biofilms could be observed when viewing biofilms using confocal microscopy (Fig. S2).

In mixed-strain populations, such as those in CF patients, Δ*lasI* mutants can utilize WT-produced C12-HSL to restore a functional QS system [12,31]. Autoinducer supplementation restored Δ*lasI* biofilm mass (CV staining) and live-cell numbers (c.f.u. ml^−1^) to WT levels (Fig. 3). Restoring QS may optimize autolysis and cell density regulation in Δ*lasI* mutants without the metabolic costs of autoinducer production [12,31]. By contrast, the signal-blind Δ*lasR* mutant cannot benefit from the WT-produced signal.

We applied antibiotic concentrations based on *P. aeruginosa* planktonic MICs for *P. aeruginosa* [42] and 10-fold higher (Fig. 4). Under both conditions, QS mutant strains exhibited antibiotic tolerance comparable to the WT. We suggest that the increased biomass in the QS mutant biofilms may have hindered antibiotic penetration, protecting the live cells within [22,55]. Given that these QS mutants naturally occur within patients, it is concerning that these mutants can form functional biofilms and exhibit WT-level antibiotic resistance.

Autoinducer supplementation allowed Δ*lasI* to maintain higher pre-treatment and post-treatment c.f.u. ml^−1^ compared to non-supplemented Δ*lasI* biofilms (Fig. 4). Since C12-HSL supplementation mimics the conditions of Δ*lasI* growth in the mixed-strain populations found *in vivo*, this suggests Δ*lasI* may gain an advantage over the WT and signal-blind QS mutants during treatment. Targeting *lasI* mutants with QS inhibitors could eliminate this advantage in mixed-strain infections [14,31, 52].

While we only examined biofilm survival up to 72 h, Δ*lasI* and Δ*lasR* mutations may affect biofilm viability and growth differently in more mature biofilms [12,36]. A study examining 2-week-old biofilms found that Δ*lasI* biofilms were 20% thinner than the WT and prone to detachment when exposed to the detergent sodium dodecyl sulphate [53]. However, this disadvantage of Δ*lasI* may be mitigated in the ecological context of mixed-strain communities where these QS mutants evolve [12,34].

The molecular mechanisms that underpin the observed autolysis phenotypes of *P. aeruginosa* QS mutants remain unclear and an exciting area for future study. As biofilms of *P. aeruginosa* are responsible for persistent and difficult-to-treat infections in immunocompromised patients and clinical settings, research to elucidate the control and consequences of autolysis, and the role of prophages, holds exciting potential for developing novel antimicrobial therapies.

## Supplementary material

10.1099/mic.0.001557 Uncited Supplementary Material 1.

## Abbreviations:

AMR: antimicrobial resistance
CF: cystic fibrosis
c.f.u.: colony-forming units
C12-HSL: *N*-3-oxododecanoyl homoserine lactone
CV: crystal violet
eDNA: extracellular DNA
LB: Luria-Bertani
MICs: minimum inhibitory concentrations
OD: optical density
PBS: phosphate-buffered saline
QS: quorum sensing
WT: wild-type
