# Supplementary material for: Disruption of Pseudomonas aeruginosa quorum sensing influences biofilm formation without affecting antibiotic tolerance
Source: Microbiology (Reading). 2025 Apr 25;171(4):001557. doi: 10.1099/mic.0.001557 (PMC12032407; doi:10.1099/mic.0.001557)
Supplement: Uncited Supplementary Material 1. [file mic-171-01557-s001.pdf]

Disruption of *Pseudomonas aeruginosa* quorum-sensing influences biofilm formation without affecting antibiotic tolerance

Elvina Smith[1], Andrew Matthews[1], Edze R Westra[1*] & Rafael Custodio[1,2,3*]

[1]Environment and Sustainability Institute, Biosciences, University of Exeter, Penryn Campus, Penryn TR10 9FE, UK

[2]IBMC, Instituto de Biologia Molecular e Celular, Universidade do Porto, Porto, Portugal.

[3]i3S, Instituto de Investigação e Inovação em Saúde, Universidade do Porto, Porto, Portugal.

*These authors contributed equally to this work

Correspondence to: r.c.da-silva-custodio@exeter.ac.uk; E.R.Westra@exeter.ac.uk

SUPPLEMENTARY MATERIAL

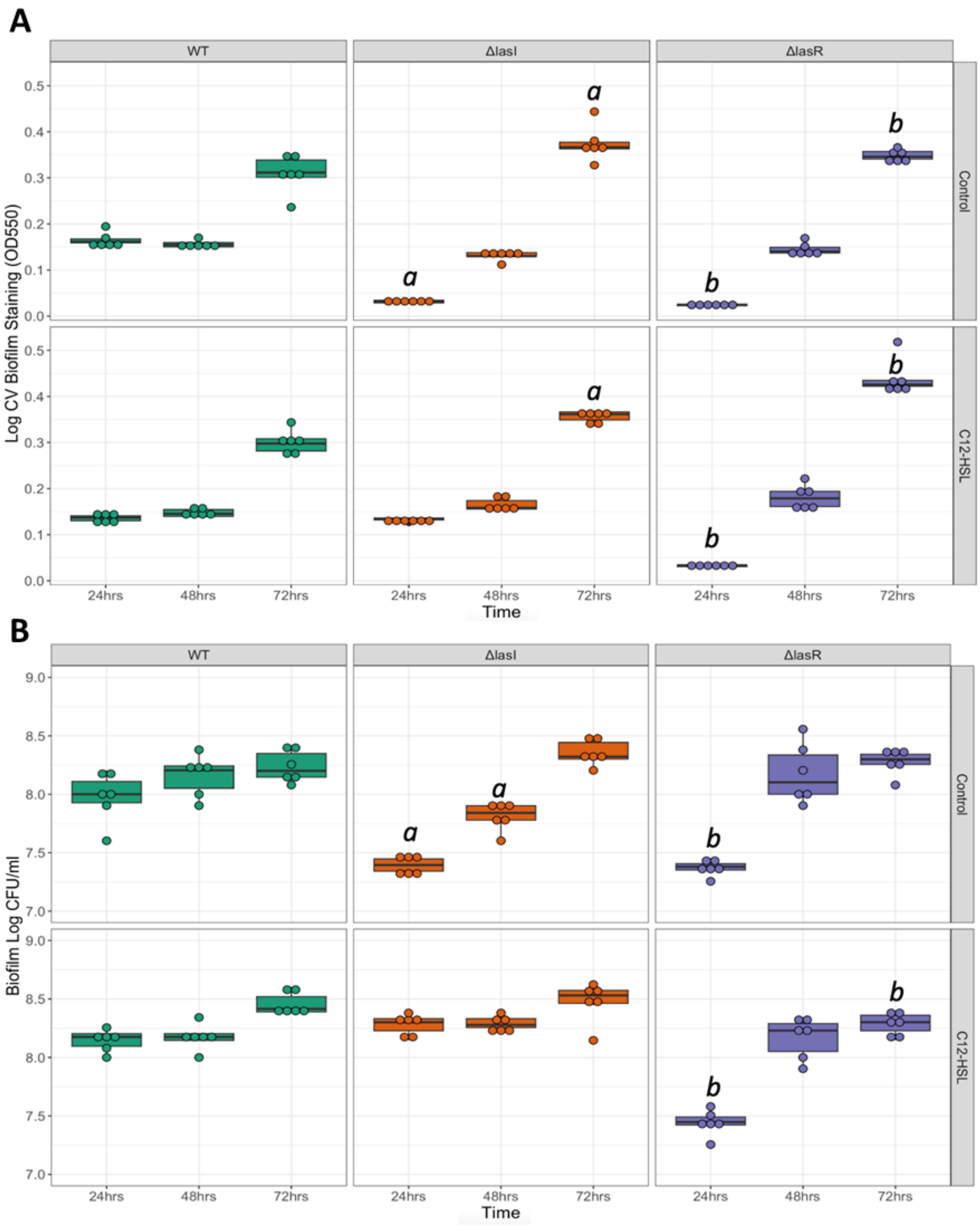

Supplementary Figure 1: Biofilm formation of *Pseudomonas aeruginosa* PA14 wild-type (WT) and quorum-sensing (QS) mutants (Δ*lasI* and Δ*lasR*) with and without autoinducer supplementation, with media replenished daily; Measured by crystal violet (CV) staining and colony forming units (CFU/ml). Biofilms of each strain were grown in wells of microtiter plates, for 24, 48 and 72 hours. C12-HSL wells were supplemented with C12-HSL autoinducer at a 2mM/ml concentration. Biofilms received daily fresh media, with the old media removed and replaced with new M63 medium each day. Box plots show the median

and interquartile range, with individual data points plotted (N=6 replicates per strain, time point and treatment). Values are presented on the $\log_{10}$ scale. Significant differences (Tukey HSD: $P<0.05$) identified between the WT and QS mutants at individual growth times are marked with *a* for Δ*lasI* and *b* for Δ*lasR.* **A)** Biofilms were stained using crystal violet (CV) and the optical density (OD) of solubilised crystal violet measured at 550nm. **B)** Colony forming units (CFU/ml) recovered from biofilms.

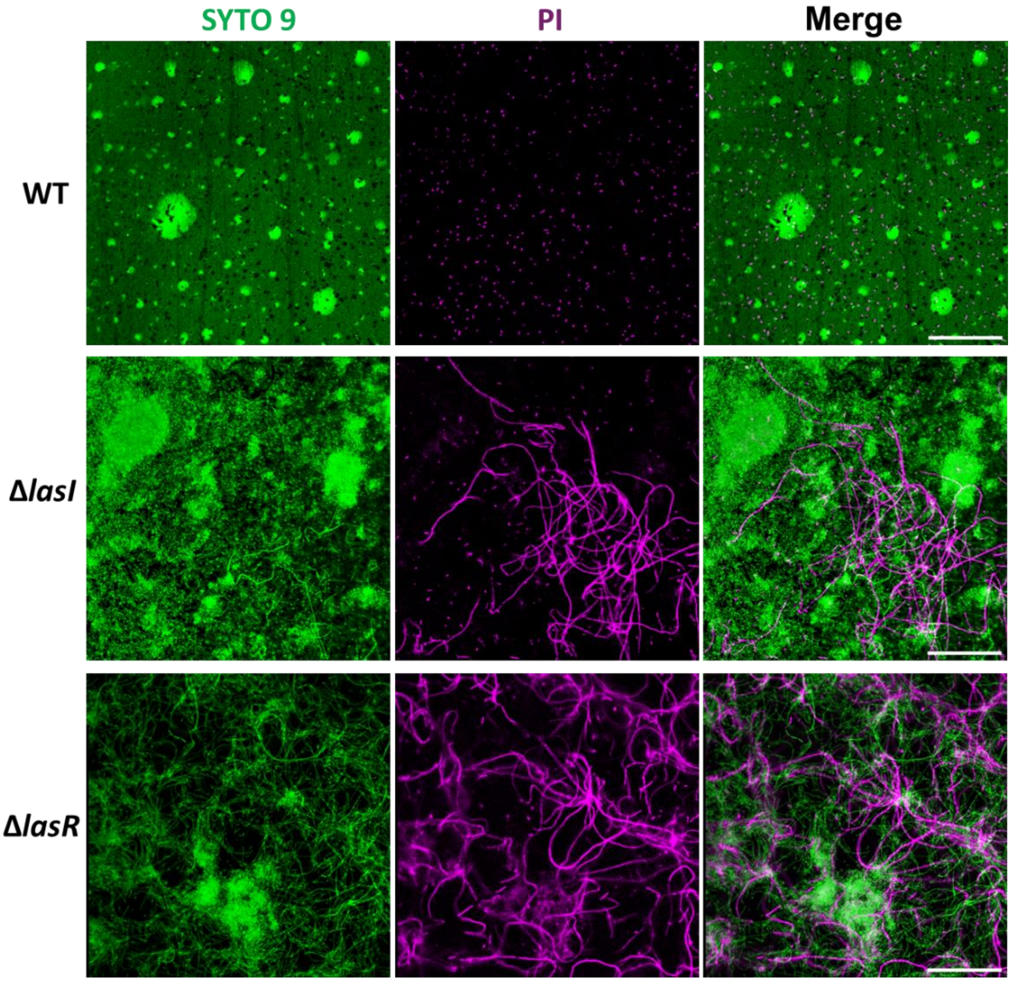

Supplementary Figure 2. QS mutants display increased cell death and filamentation during biofilms formation. 24 h-old biofilms of *P. aeruginosa* and QS mutants were analysed by confocal microscopy. After 24 h incubation, bacterial viability was assayed by staining the cells with *Bac*Light bacterial viability kit: Propidium iodide (PI; magenta areas are dead

bacteria) and Syto 9 (green areas are live bacteria). Images are from a single plane to depict staining pattern. Scale bar corresponds to 100 uM.
